# Discontinuation or reduction in benzodiazepine use by treatment with the traditional herbal medicine Hangekobokuto, case reports

**DOI:** 10.1002/jgf2.313

**Published:** 2020-04-01

**Authors:** Ryo Yoshinaga, Toshinobu Maki, Yusuke Goto, Hiroki Inoue, Hiromi Yano, Eiichi Tahara

**Affiliations:** ^1^ Department of Japanese Oriental Medicine Iizuka Hospital Iizuka City Japan

**Keywords:** effect of medication, elderly, polypharmacy, use of medication

## Abstract

In this report, we present two cases in which benzodiazepines (BZDs) were able to be successfully reduced or discontinued by treatment with traditional Japanese herbal medicine (Kampo medicine), including Hangekobokuto (HKT). These two patients with long‐term use of BZDs due to mental disorders suffered epigastric symptoms. After starting Kampo therapy including HKT, their epigastric symptoms were improved and they were able to reduce or discontinue the use of BZDs. This suggests that HKT is a potentially promising substitutive pharmacotherapy for patients with long‐term use of BZDs. HKT should be considered as a treatment for patients with mental disorders who have taken BZDs for a long time and suffer from medically unexplained epigastric symptoms.

## Abstract 2

In this report, we present two cases in which benzodiazepines were able to be successfully reduced or discontinued by treatment with traditional Japanese herbal medicine (Kampo medicine), including Hangekoubokutou (HKT). HKT is a potentially promising substitutive pharmacotherapy for patients with long‐term use of benzodiazepines.

## 1 INTRODUCTION

Benzodiazepines (BZDs) are widely prescribed to treat anxiety and sleep disorders, and over‐prescription of BZDs is often seen in primary care settings.^1^ A Screening Tool of Older Persons’ Prescriptions version 2 stated that BZDs for older patients be prescribed for no more than 4 weeks.^2^ Long‐term use of BZDs is associated with not only physical dependence^3^ but also various harmful effects including fractures,^4^ car accidents,^5^ and cognitive impairment.^6^ We experienced two cases in which BZDs were successfully reduced or discontinued by treatment with traditional Japanese herbal medicine, including Hangekobokuto (HKT).

## 2 CASE 1

The patient was a 75‐year‐old man who complained of appetite loss due to throat and epigastric discomfort. His medical history included hypertension and endoscopic surgery for esophageal cancer. Six months previous, he had feelings of stagnation between his throat and epigastric area. He lost his appetite and 4 kg of body weight. He could not eat rice but could eat only watery cooked rice and udon. The patient underwent various examinations including blood testing, upper gastrointestinal endoscopy, abdominal ultrasonography, abdominal CT examination, and cardiac catheterization in a number of hospitals, with no abnormal results. His sense of stagnation was relieved when etizolam (0.25 mg) was administered twice or three times a day. He was diagnosed with somatic symptom disorder by doctors in the General Internal Medicine and Psychosomatic Medicine departments. The patient denied that he had psychological problems. He hoped for Kampo therapy and elected to visit our department. His height was 160 cm, weight 50 kg, blood pressure 133/78 mm Hg, and heart rate 110 beats/min. He had resistance and tenderness in the epigastrium. He reported anxiety, fatigue, abnormal sensation in his throat, chest stuffiness, appetite loss, indigestion, and burping. Based on these symptoms, his questionnaire for diagnosis of Qi stagnation^7^ was calculated as 82.67. At the first visit, HKT decoction was administered. Three weeks later, his symptoms disappeared, he felt better, and he could eat rice. Eight weeks later, he gained 1 kg and discontinued the use of etizolam. One year later, his questionnaire for diagnosis of Qi stagnation decreased to 9.30. The patient continued taking HKT for two and a half years without adverse effects. He remained healthy with a weight of 53 kg.

## 3 CASE 2

The patient was a 70‐year‐old man who complained of heartburn and stomach discomfort. His medical history included depression when he was 65 years old. He was prescribed etizolam (0.5 mg), estazolam (2 mg), clonazepam (1.5 mg), clomipramine (10 mg), and ranitidine (150 mg) by a psychiatrist for several years, and he had suffered from heartburn and stomach discomfort during this period. The psychiatrist recommended Kampo therapy to him because his epigastric symptoms and depressive mood were intractable, and he was voluntarily referred to our department. His height was 156 cm, weight 54 kg, blood pressure 122/80 mm Hg, and heart rate 64 beats/min. He had epigastric discomfort and resistance. He reported anxiety, headache, abnormal sensation in his throat, chest stuffiness, a feeling of oppression in the costal region, abdominal fullness, appetite loss, and indigestion. Based on these symptoms, his questionnaire for diagnosis of Qi stagnation^7^ was calculated as 108.37. At the first visit, 7.5 g/d Bukuryoingohangekobokuto extract, which combines Bukuryoin and Hangekobokuto, was administered. One month later, his stomach discomfort was relieved. Two months later, he discontinued clomipramine (10 mg), clonazepam (1.5 mg), and ranitidine (150 mg) of his own accord and was able to resume playing table tennis which he had enjoyed in the past. One year and 4 months later, he suffered from pneumonia and was treated in hospital. After his pneumonia improved, he reported appetite loss and general fatigue. His Kampo therapies were changed to 5.0 g/d Ninjinto (NJT) extract and 5.0g/day HKT extract. Two and a half years later, the patient discontinued the use of etizolam, leaving estazolam (2 mg) as his only western medical treatment. Three years later, his questionnaire for diagnosis of Qi stagnation decreased to 26.95. The patient continued taking NJT and HKT for four years without adverse effects and remained healthy.

## 4 DISCUSSION

Benzodiazepines were able to be reduced or discontinued in two patients with mental disorders by treating them with Japanese herbal medicine, including HKT. Alternative drugs such as melatonin, paroxetine, and trazodone were shown to be effective in the cessation of BZDs in a previous systematic review.^8^ However, evidence for the use of alternative pharmacotherapy in the management of BZDs remains relatively scarce. To the best of our knowledge, this is the first report to show that HKT could be a useful substitutive medication for patients with long‐term use of BZDs.

Hangekobokuto is one of the most basic compounds in traditional Chinese medicine and has been widely used as a representative drug for the treatment of Qi stagnation. Qi, which corresponds to spirit, energy, and gas, means invisible action, function, or working that circulates throughout the body.^9^ Qi stagnation is the condition in which circulation of Qi is disturbed and is often encountered in today's stressful society. Many patients with Qi stagnation complain of a depressive mood, an abnormal sensation in the throat, and gas retention. A questionnaire for diagnosis of Qi stagnation was developed in a previous study.^7^ The study estimated that its sensitivity and specificity were 0.83 and 0.80, respectively. The questionnaire analyzes 23 symptoms. A total score of more than 28.5 points is considered significant for Qi stagnation. The questionnaire evaluates digestive symptoms and anxiety as a psychogenic factor at the same time. These two cases could be diagnosed as Qi stagnation using this diagnostic method, and their scores decreased over time after initiating Kampo therapy.

An abnormal sensation in laryngopharynx is the most common target symptom to prescribe HKT. Digestive symptoms, especially in the epigastric area, are also a good indication for HKT. A previous study demonstrated that HKT improves gastrointestinal symptoms in functional dyspepsia patients by reducing bowel gas.^10^ In addition, both cases had objective resistance of the epigastric region on pressure in Kampo abdominal examination, which is an objective finding for prescribing HKT.^9^ Some animal studies have also shown that HKT has an antidepressant effect.^11^, ^12^ Since HKT can be expected to alleviate epigastric and psychological problems concurrently, patients with long‐term use of BZDs who have epigastric symptoms may be good candidates for Kampo therapy.

Furthermore, the patient in Case 1 ceased receiving repeated medical examinations and the patient in Case 2 discontinued use of an antidepressant drug and an H2 blocker by initiating Kampo therapy. Therefore, Kampo therapy may contribute to solving the problems of doctor shopping and polypharmacy.

This report has some limitations. Firstly, placebo effect may influence these cases. Further clinical trials are necessary to establish the efficacy of HKT in patients with long‐term use of BZDs.

In conclusion, Kampo therapy, especially that including HKT, is a possible alternative treatment for patients with long‐term use of BZDs. HKT may be useful for discontinuation or reduction in BZDs use and should be considered for patients with mental disorders who suffer from medically unexplained epigastric symptoms in terms of western medicine.

## 5 ADDITIONAL NOTE

The HKT decoction in Case 1 consisted of Pinellia tuber 8.0 g, Poria Sclerotium 5.0 g, Magnolia Bark 3.0 g, Perilla Herb 2.0 g, and Ginger 1.0 g. The decoction was prepared by boiling the crude drugs in 600 mL of water for 40 minutes until the volume of the solution was concentrated to 300 mL. The decoction was administered 3 times a day. All extracts in Case 2 were prepared by Tsumura Company, Tokyo, Japan.

## CONFLICT OF INTERESTS

Ryo Yoshinaga and Eiichi Tahara have received lecture fees from Tsumura & Co.

## AUTHOR CONTRIBUTIONS

RY performed clinical assessments. TM, YG, HI, H.Y, and ET were involved in revising the manuscript. RY wrote the manuscript. All authors significantly contributed to data interpretation and manuscript preparation. All authors read and approved the final version of the manuscript.

## INFORMED CONSENT

We have obtained written informed consent from the patients for publication of this case report.

## References

[1] Davies J , Rae TC , Montagu L . Long‐term benzodiazepine and Z‐drugs use in England: a survey of general practice [corrected]. Br J Gen Pract. 2017;67(662):e609–13. 28716996 10.3399/bjgp17X691865 PMC5569740

[2] O'Mahony D , O'Sullivan D , Byrne S , O'Connor MN , Ryan C , Gallagher P . STOPP/START criteria for potentially inappropriate prescribing in older people: version 2. Age Ageing. 2015;44(2):213–8. 25324330 10.1093/ageing/afu145 PMC4339726

[3] Tyrer P . Risks of dependence on benzodiazepine drugs: the importance of patient selection. BMJ. 1989;298(6666):102–5. 2493275 10.1136/bmj.298.6666.102 PMC1835446

[4] Sorock GS , Shimkin EE . Benzodiazepine sedatives and the risk of falling in a community‐dwelling elderly cohort. Arch Intern Med. 1988;148(11):2441–4. 2903726

[5] Thomas RE . Benzodiazepine use and motor vehicle accidents. Systematic review of reported association. Can Fam Physician. 1998;44:799–808. 9585853 PMC2277821

[6] Stewart SA . The effects of benzodiazepines on cognition. J Clin Psychiatry. 2005;66(Suppl 2):9–13. 15762814

[7] Okitsu R , Iwasaki K , Monma Y , Takayama S , Kaneko S , Shen G , et al. Development of a questionnaire for the diagnosis of Qi stagnation. Complement Ther Med. 2012;20(4):207–17. 22579432 10.1016/j.ctim.2011.12.005

[8] Parr JM , Kavanagh DJ , Cahill L , Mitchell G , Mc DYR . Effectiveness of current treatment approaches for benzodiazepine discontinuation: a meta‐analysis. Addiction. 2009;104(1):13–24. 10.1111/j.1360-0443.2008.02364.x 18983627

[9] Health and Labour Sciences Research Grants .Textbook of Traditional Japanese Medicine, Part 1: Kampo. Report to the Ministry of Health, Labour and Welfare for 2012, Health and Labour Sciences Research Grants 2012;68–101.

[10] Oikawa T , Ito G , Hoshino T , Koyama H , Hanawa T . Hangekobokuto (Banxia‐houpo‐tang), a Kampo medicine that treats functional dyspepsia. Evid Based Complement Alternat Med. 2009;6(3):375–8. 18955239 10.1093/ecam/nem101 PMC2722198

[11] Li JM , Kong LD , Wang YM , Cheng CH , Zhang WY , Tan WZ . Behavioral and biochemical studies on chronic mild stress models in rats treated with a Chinese traditional prescription Banxia‐houpu decoction. Life Sci. 2003;74(1):55–73. 14575813 10.1016/j.lfs.2003.06.030

[12] Li JM , Yang C , Zhang WY , Kong LD . The effects of banxia houpu decoction on a chronic mild stress model of depression. Zhongguo Zhong Yao Za Zhi. 2003;28(1):55–9. 15015269

